# Development and validation of the Diabetes Numeracy Test (DNT)

**DOI:** 10.1186/1472-6963-8-96

**Published:** 2008-05-01

**Authors:** Mary Margaret Huizinga, Tom A Elasy, Kenneth A Wallston, Kerri Cavanaugh, Dianne Davis, Rebecca P Gregory, Lynn S Fuchs, Robert Malone, Andrea Cherrington, Darren A DeWalt, John Buse, Michael Pignone, Russell L Rothman

**Affiliations:** 1 Division of General Internal Medicine and Public Health, Department of Medicine, Center for Health Services Research, Vanderbilt University Medical Center, Nashville, USA; 2 VA Quality Scholars Program, VA Tennessee Valley Healthcare System, Nashville, USA; 3 Diabetes Research and Training Center, Vanderbilt University Medical Center, Nashville, USA; 4 School of Nursing, Vanderbilt University Medical Center, Nashville, USA; 5 Division of Nephrology, Department of Medicine, Vanderbilt University Medical Center, Nashville, USA; 6 Peabody College of Education, Vanderbilt University, Nashville, USA; 7 Department of Medicine, University of North Carolina, Chapel Hill, USA; 8 Department of Medicine, University of Alabama, Birmingham, USA

## Abstract

**Background:**

Low literacy and numeracy skills are common. Adequate numeracy skills are crucial in the management of diabetes. Diabetes patients use numeracy skills to interpret glucose meters, administer medications, follow dietary guidelines and other tasks. Existing literacy scales may not be adequate to assess numeracy skills. This paper describes the development and psychometric properties of the Diabetes Numeracy Test (DNT), the first scale to specifically measure numeracy skills used in diabetes.

**Methods:**

The items of the DNT were developed by an expert panel and refined using cognitive response interviews with potential respondents. The final version of the DNT (43 items) and other relevant measures were administered to a convenience sample of 398 patients with diabetes. Internal reliability was determined by the Kuder-Richardson coefficient (KR-20). An *a priori *hypothetical model was developed to determine construct validity. A shortened 15-item version, the DNT15, was created through split sample analysis.

**Results:**

The DNT had excellent internal reliability (KR-20 = 0.95). The DNT was significantly correlated (p < 0.05) with education, income, literacy and math skills, and diabetes knowledge, supporting excellent construct validity. The mean score on the DNT was 61% and took an average of 33 minutes to complete. The DNT15 also had good internal reliability (KR-20 = 0.90 and 0.89). In split sample analysis, correlations of the DNT-15 with the full DNT in both sub-samples was high (rho = 0.96 and 0.97, respectively).

**Conclusion:**

The DNT is a reliable and valid measure of diabetes related numeracy skills. An equally adequate but more time-efficient version of the DNT, the DNT15, can be used for research and clinical purposes to evaluate diabetes related numeracy.

## 

In 1992, the National Adult Literacy Survey (NALS) estimated that over 90 million adult Americans have inadequate literacy and numeracy skills [1]. More recently, the National Assessment of Adult Literacy (NAAL) showed that 36% of American adults had basic or below basic health-related literacy and quantitative skills [2]. In that survey, 22% of adults demonstrated below basic quantitative skills and could only perform simple, concrete, single-step math operations, such as addition. Another 33% demonstrated basic quantitative skills, solving single-step math problems where the operation was either given or easily inferred. Patients with low literacy can have difficulty following medical instructions, understanding health information and performing self-management tasks [3]. Low literacy is associated with worse disease knowledge and poorer clinical outcomes in patients with diabetes [4,5]. The broad construct of literacy consists of several components: print literacy, oral literacy and quantitative skills [6]. Many of the recent studies evaluating the relationship between literacy and health have focused on print literacy with little attention given to quantitative or numeracy related skills. While common for patients with low literacy to have inadequate quantitative skills, patients with adequate literacy may still have difficulty with numeracy.

Numeracy is an important component of literacy that can be simply defined as the ability to understand and use numbers in everyday life [6-10]. Numeracy does not refer solely to basic mathematical skills (arithmetic, calculations, fractions, algebra and geometry) but also to one's ability to understand time, currency, measurement, graphic representations, logic, hierarchies and probability. To apply numeracy to a specific problem or task, one must know which numerical skill(s) to apply, deduce and interpret the result(s) and then appropriately apply the result(s) to the situation [7,11]. For a patient with diabetes, numeracy is needed to interpret blood glucose meter data, properly administer medications and follow nutritional recommendations. For example, a patient with diabetes may need numeracy to calculate their carbohydrate intake and adjust their insulin based on carbohydrates and/or current blood glucose level. Poor numeracy skills in patients with diabetes could lead to suboptimal glycemic control, increased hypoglycemic episodes or widely varying glucose values.

Scales have been developed to measure literacy in the health care setting [12-14]. One of these scales, the TOFHLA, did include 17 items to measure general quantitative skills [14]. These items primarily focused on reading prescriptions and other materials that tested simple mathematical skills such as understanding dates and timing of medication dosage. These items were highly dependent on reading ability and, not surprisingly, they are highly correlated to reading ability. These numeracy items are not included in the shortened form, the S-TOFHLA [15]. The TOFHLA was not designed to measure the range of numeracy skills needed in diabetes management and does not give a provider clinically useful information in the care of a patient with diabetes [10].

There are no current scales designed specifically to assess diabetes related numeracy skills required to perform daily diabetes self-management. Numeracy skills may be particularly critical in the management of diabetes and the currently available health literacy scales may not adequately identify patients with low numeracy. In order to better understand numeracy in diabetes, the Diabetes Numeracy Test (DNT) was developed. The DNT was specifically designed to evaluate the wide range of numeracy skills used by patients with diabetes.

## Research design and methods

### Scale development

The first phase of development included item generation by a group of experts in diabetes, literacy and numeracy. This group included diabetologists, certified diabetes educators, primary care providers, registered dietitians, behavioral researchers in diabetes, and literacy and numeracy experts. The initial item pool contained 70 items that addressed all mathematical skills required in the daily management of diabetes. Those 70 items covered five diabetes self-management areas (nutrition, exercise, glucose monitoring, oral medication and insulin use) and the general mathematical skills required in the management of diabetes including addition, subtraction, multiplication, division, fractions/decimals, numerical hierarchy, inference skills and multi-step calculations. The 70 items were administered to 40 individuals without diabetes to assess understandability. To eliminate redundancy, the expert panel reduced the measure to 45 items that represented the five self-management areas. The panel agreed that the 45 item scale had adequate content validity to address the range of numeracy skills required in the management of diabetes.

The objective of the second phase of development of the DNT was to address the clarity of items for patients with diabetes. Ten cognitive response interviews were conducted with patients with diabetes to assess each item. Interviewees were asked specific questions about each item to assess the understandability of the wording. If an item was unclear, the interviewee was told the purpose of the item and then encouraged to suggest an alternate format or wording. In response to the interviews, the scale was reformatted and slightly reduced to the final 43 items [16].

The DNT was designed to assess nutrition, exercise, glucose monitoring, oral medication and insulin skills that patients may encounter during daily diabetes self-management. There are nine nutrition items focusing on nutrition label interpretation and carbohydrate counting. Four exercise items assess carbohydrate intake and insulin adjustment for exercise time. Blood glucose monitoring skills are evaluated by four items about number hierarchy, glycated hemoglobin and calculating supplies needed. Five items assess oral medication use, refill patterns and dates, and oral titration schemes. The remaining 21 items determine numeracy associated with insulin use including interpretation of syringes, correction or sliding scale insulin use, insulin adjustment for carbohydrate intake and titration instructions. See Table 1 for a description of the diabetes care domains and numeracy skills assessed in the DNT by item number. Items are scored as binary outcomes – correct or incorrect – and no partial credit is given. There is no time limit for the administration of the scale. Many patients with diabetes use calculators; therefore, participants were allowed to use calculators during the administration of the DNT to emulate real-life circumstances. DNT scores are reported as percent correct (with a possible range of 0% to 100%).

**Table 1:** Description of DNT Items.

Diabetes Care Domain	Question Number
Nutrition	1–9
Exercise	10–13
Blood Glucose Monitoring	14–17
Oral Medication Use	18–22
Insulin Use	23–43

Numeracy Domain	Question Number

Addition	2,25
Subtraction	8
Multiplication	3,5,16,26,27
Division	11,21,28–31
Fractions/Decimals	4,6,7,8
Multi-step mathematics	9,12,13,20,35–43
Time	10,17,22
Numeration/Counting/Hierarchy	1,14,15,18,19,23,24,32–34

**Figure 1:**
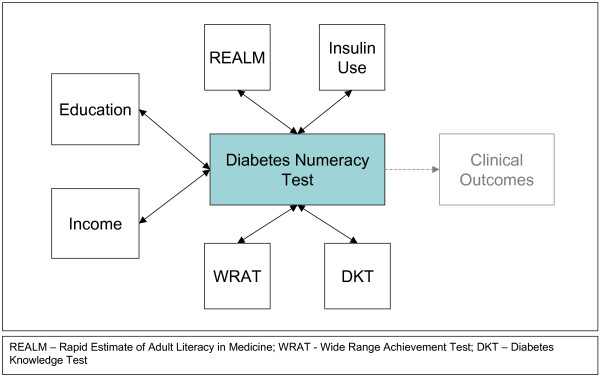
**DNT a *priori *Construct Validity Model.** There is no gold standard for diabetes numeracy skills, therefore construct validity was used. This *a priori *model of correlations was determined by the expert panel to assess construct validity of the DNT.

Phase 3 of development sought to assess the reliability and provide support for the construct validity of the DNT. Reliability was evaluated through internal consistency testing with the Kuder-Richardson 20 formula. There is no gold standard for numeracy in diabetes; hence an *a priori *model of correlations was determined by the expert panel to assess construct validity (see Figure 1). We hypothesized that increased education, income, literacy, math skills, and diabetes knowledge would all be moderately associated (rho of 0.3 – 0.6) with improved DNT scores. We also hypothesized that insulin use would be moderately associated with higher DNT since many of the DNT questions focused on insulin administration.

In the final phase of the study, the scale was shortened to a more clinically useful 15 items and then verified through random split sample analysis. To perform the split sample analysis, the sample data were randomly split into two smaller sub-samples. Sub-sample 1 was used for the development of the shortened scale and sub-sample 2 was used for confirmation of the results. In order to determine the items to include in the DNT15, principal components analysis was performed on sub-sample 1. Items with > 0.6 loading on the primary component in sub-sample 1 were included. In addition, those items with > 80% mean correct score were discarded. Three items with high face validity, as determined by practicing diabetologists and diabetes educators, were added back in to bring the total number of items to 15. Reliability was evaluated by internal consistency (Kuder-Richardson 20) and validity was assessed through correlation testing using Spearman's correlations between the DNT15 and the full DNT and comparing the DNT15 to the *a priori *construct validity model for both sub-samples.

### Participant selection

Participants in this study were recruited from four sites: general medicine clinics at two academic health centers, a diabetes clinic at an academic health center and an endocrinology clinic at a VA health center. Participants were paid $20 to participate. A convenience sample of 398 participants was recruited at clinic visits. Inclusion criteria included diagnosis of type 1 or 2 diabetes, age 18 to 80 years and English speaking. Potential participants were excluded if their corrected visual acuity was > 20/50 using a Rosenbaum Pocket Vision Screener or if they had a diagnosis of significant dementia, psychosis or blindness.

### Measures

Trained research assistants collected patient characteristics, blood glucose monitoring frequency data from glucose meters, and administered a series of surveys to the patients. Measures included a validated literacy measure (REALM), a validated general numeracy scale (WRAT-3R) and a validated diabetes knowledge test (DKT) [12,17,18].

### Statistical analysis

Internal consistency was determined by the Kuder-Richardson 20 coefficient of reliability. Spearman's rank order correlations were performed for the *a priori *model testing. Principal component analysis with oblique rotation was performed to determine the presence of multiple factors and item loadings on the factors. For development of the DNT15, Spearman's correlations were used in the random split sample analysis. Stata 9.2 (StataCorp, College Station, TX) was used for all statistical analysis.

All work was approved by the institutional review boards of Vanderbilt University and the University of North Carolina.

## Results

A group of 398 patients with diabetes completed the DNT and the construct validation scales including the WRAT, REALM and DKT. Patient characteristics are presented in Table 2. The average age was 54.2 years and 51% of the participants were female. Eighty-six percent had type 2 diabetes. The majority (83%) of participants had completed high school but only 69% had greater than 9^th ^grade literacy by the REALM and only 31% had greater than 9^th ^grade numeracy by the WRAT. The 43-item DNT took an average (± SD) of 33 ± 13 minutes to complete. The average score (± SD) on the DNT was 61% ± 25.

**Table 2:** Patient Characteristics (n = 398).

Characteristic	Mean ± SD or n (%)
Age	54.2 ± 13
Race	
White	249 (63)
Black	134 (34)
Other	14 (3)
Sex	
Male	196 (49)
Female	202 (51)
Annual Family Income	
<$20,000	171 (44)
$20,000-$40,000	95 (24)
$40,000-$60000	58 (15)
≥ $60,000	66 (17)
Recruitment site	
VA diabetes center	65 (16)
Academic center primary care	200 (50)
Academic diabetes clinic	133 (33)
Education	
<High School	64 (16)
High School or GED	103 (26)
Some college	115 (29)
College or more	110 (28)
Literacy status, REALM	
≤ 6^th ^grade	33 (8)
7^th^-8^th ^grade	92 (23)
≥ 9^th ^grade	273 (69)
Numeracy status, WRAT	
≤ 6^th ^grade	211 (53)
7^th^-8^th ^grade	65 (16)
≥ 9^th ^grade	122 (31)
DKT	0.69 ± 0.18
BMI, m/kg^2^	33.6 ± 8.1
Type of diabetes	
Type 1	57 (14)
Type 2	341 (86)
On insulin, n = 240	240 (60)
Adjusts for blood glucose	141 (57)
Adjusts for carbohydrate intake	90 (36)
Blood glucose monitoring	
≤ 1 time per day 2–3 times per day	133 (48) 185 (46)
≥ 4 times per day	80 (20)
Received prior diabetes education	331 (83)
Hemoglobin A1c, (%)	7.6 ± 1.7
Duration of diabetes, years	11 ± 9

GED – General Education Development Test; REALM – Rapid Estimate of Adult Literacy in Medicine;

WRAT – Wide Range Achievement Test; DKT – Diabetes Knowledge Test; BMI – Body Mass Index (kg/m^2^)

**Figure 2:**
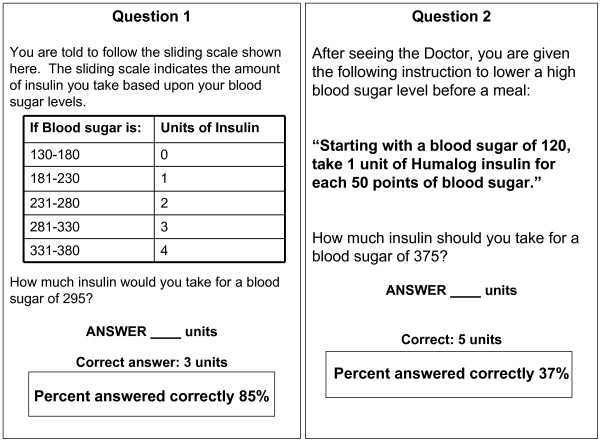
**DNT Sample Questions.** Two commonly used methods for sliding scale insulin adjustment instructions are displayed. Participants were able to correctly answer Question 1 more often than Question 2.

Problem areas for participants included titration schemas, food label interpretation, insulin adjustment instructions, dates for refills and items that required multi-step math (e.g., calculating insulin dosage based on carbohydrate intake and glucose level). Figure 2 illustrates the importance of how the framing of diabetes education can impact patient performance. Two commonly used methods for sliding scale insulin adjustment instructions are displayed. Question 1, with a simple table to interpret, was answered correctly by 85% of participants in this study. However, question 2, which required patients to interpret a word problem and apply multiple numerical steps to determine their insulin dosage, was only answered correctly by 37% of the participants.

The 43-item version of the DNT is highly reliable as determined by internal consistency (KR-20 = 0.95). Construct validity was examined through the *a priori *construct model and expected correlations as determined by the expert panel (see Table 3). The DNT was moderately correlated with education, income and literacy (REALM) and strongly correlated with numeracy (WRAT-3R) and diabetes knowledge (DKT). One portion of the model did not correlate as expected. The *a priori *model predicted a moderate correlation with insulin use; however, minimal correlation with insulin use was observed (See Table 3). The DNT was also significantly correlated with frequency of blood sugar testing (rho = 0.15, p = 0.0025), patient adjustment of insulin for carbohydrate intake (rho = 0.51, p < 0.0001), and insulin adjustment for blood glucose (rho = 0.28, p < 0.0001)). Principal component analysis revealed one primary factor that accounted for 65% of the total test variance.

**Table 3:** DNT correlations with *a priori *model

Variable	Spearman's correlation (rho)	P value
Education	0.52	<0.0001
Income	0.51	<0.0001
REALM	0.54	<0.0001
WRAT	0.62	<0.0001
DKT	0.71	<0.0001
Insulin use	0.04	0.4313

REALM – Rapid Estimate of Adult Literacy in Medicine; WRAT – Wide Range Achievement Test; DKT – Diabetes Knowledge Test

The DNT15 showed similar internal consistency and validity in both sub-samples as the full DNT. The KR-20 of the DNT15 was 0.90 in the development sample (sample 1) and 0.89 in confirmation sample (sample 2). Correlations with the *a priori *model were similar to the full DNT in both population sub-samples (see Table 4). The DNT15 was highly correlated with the full DNT (see Table 4). In a separate study, the approximate time of administration is 10–15 minutes.

**Table 4:** DNT15 and sub-sample analysis: Spearman's correlations.

Variable	Sub-sample 1 (rho)	Sub-sample 2 (rho)
DNT	0.96	0.97
Education	0.36	0.52
Income	0.53	0.49
REALM	0.54	0.52
WRAT	0.65	0.61
DKT	0.73	0.67
Insulin use	0.10	-0.02

DNT – Diabetes Numeracy Test; REALM – Rapid Estimate of Adult Literacy in Medicine; WRAT – Wide Range Achievement Test; DKT – Diabetes Knowledge Test

## Discussion

Psychometric analysis of the 43 item version of the DNT shows that it has good reliability and validity in testing numeracy skills in patients with diabetes. Of the six *a priori *construct hypotheses, five demonstrated significant correlations in the expected directions. The DNT was correlated with diabetes knowledge, education, socioeconomic status, literacy and general numeracy. Insulin use was not correlated with the DNT scores as initially predicted. This is likely due to the fact that many patients on insulin may be placed on long-acting insulin in which one or two doses may be administered with no adjustment for blood glucose or carbohydrate intake. When complexity of the insulin regimen was taken into account, more complex regimens including adjustment for carbohydrate intake and blood glucose level were significantly associated with the DNT.

The shortened version, the DNT15, also showed good reliability and construct validity. The DNT15 was designed to retain the items that discriminated diabetes related numeracy skills while also keeping the items that would be most useful to a diabetes educator or clinician. The five diabetes self-care areas are retained in the DNT15, including three items on nutrition, one item about exercise, three items regarding blood glucose monitoring, one item on oral medications and seven items about insulin administration. A diabetes educator or clinician may use the DNT15 to help target education or guide therapy.

Patients with low literacy also showed low numeracy skills on both the WRAT and the DNT. However, there were several patients with literacy skills above the ninth grade level who had low numeracy skills. Although our population was, on average, highly educated, the mean score on the DNT was only 61% correct. This suggests that numeracy should be evaluated separately from literacy. Patients with low literacy need special instructions and interventions, and patients with inadequate numeracy skills may also require modified counseling and education to improve health outcomes.

This study has several limitations. The DNT was initially developed without input from patients to determine what numeracy skills patients viewed as important. We used a convenience sample of patients recruited from academic and VA clinics, and it is possible that patients who did not participate in the study may have had different literacy and numeracy skills than those that participated. The DNT has been tested in English speaking participants only. The DNT was, in part, validated against a commonly used literacy assessment tool (the REALM), but most patients in the study scored at the highest level that the REALM assesses. A more refined assessment of reading ability at the higher levels may have been more useful.

The DNT and DNT-15 are primarily research tools for understanding the role of diabetes specific numeracy in the management of diabetes. The clinical utility of these scales will be the subject of future research. The treatment of diabetes requires the application of many literacy and numeracy skills. As the disease progresses, the complexity of the regimen may also progress. Patients need ongoing education to appropriately treat their diabetes. However, little is known about the benefits of targeting education to a patient's level of diabetes specific numeracy to improve health outcomes. Other studies have identified the role of literacy and low-literacy techniques in the improvement of health outcomes in diabetes and congestive heart failure [19,20]. Patients with low numeracy may benefit from similar interventions that address numeracy, particularly in the setting of diabetes management. The DNT and the DNT15 can provide measurement of diabetes specific numeracy and provide more information about the role of disease specific numeracy in future studies. More studies are needed to further understand the role of numeracy tailored interventions in the management of diabetes.

However, there are also clinical implications that can be learned from this study. We learned that the framing of instructions was very important in predicting patient performance. For example, study participants had a difficult time with the multi-step math required to calculate a correction dosage of insulin when instructions were presented as a sequence of sentences. This item was included to mirror clinical practice regarding how patients are currently instructed to take their insulin. Patients clearly had a much easier time calculating the insulin dosage when the material was presented in an easy to read table. This example provides an important lesson for health care providers and educators in effective communication styles for all clinical care recommendations.

## Conclusion

Numeracy, a component of literacy, is important in the daily management of many chronic diseases. Even patients with good literacy skills may have marginal numeracy skills. The full DNT and the shortened version, the DNT15, are reliable and valid scales that may be useful in identifying patients that may have deficits in diabetes related numeracy skills.

This study identified several potential problem areas for patients. These areas included food label interpretation and carbohydrate calculations, understanding of when to refill medications, understanding of insulin measurement and insulin adjustment based on blood glucose and/or carbohydrate intake, and application of medication titration instructions. Framing of the instructions impacted the understandability and may impact health outcomes. Simplification of the instructions greatly improved the participant's ability to answer the questions correctly.

To help determine the clinical utility of these scales, we have designed a new educational intervention for patients with diabetes and low literacy and numeracy skills. This new intervention is based on simple instruction, pictorial representation and color-coding for ease of use, and other accommodations to help patients, particularly those with poor literacy and numeracy skills. This intervention is currently being evaluated in a prospective randomized control trial.

## Competing interests

The authors declare that they have no competing interests.

## Authors' contributions

MMH performed the analysis of the data, developed the DNT15 and was the primary author of the manuscript. TAE and KAW participated in the development of the DNT, the design of validation study and the analysis of the data. KC assisted with the analysis of the data and the preparation of the manuscript. DD and RPG participated in the development of the DNT and in the design of the validation study. LSF provided expertise in numeracy and participated in development of the DNT. RM, AC, DD, JB and MP also participated in the development of the DNT and in the design of the validation study. RLR conceived of the study, participated in the development of the DNT, the design of the validation study, oversaw the analysis of the data, developed the DNT15, made final changes to the manuscript and provided mentorship to MMH. All authors have read and approved the final manuscript.

## Pre-publication history

The pre-publication history for this paper can be accessed here:


